# Plant NAOGAT (ArgJ) Structure Reveals Mechanism of Ornithine Biosynthesis, Preference for Acetylornithine and Susceptibility to Mangotoxin

**DOI:** 10.1111/pce.70891

**Published:** 2026-09-21

**Authors:** Maciej Nielipinski, Agnieszka J. Pietrzyk‐Brzezinska, Aleksandra Twarda‐Clapa, Bartosz Sekula

**Affiliations:** ^1^ Institute of Molecular and Industrial Biotechnology, Faculty of Biotechnology and Food Sciences Lodz University of Technology Łódź Poland

**Keywords:** arginine, arginine biosynthesis, N‐acetylornithine glutamate acetyltransferase, phytopathogens, Pseudomonas syringae, urea cycle

## Abstract

Arginine metabolism in plants is targeted by phytopathogens such as *Pseudomonas syringae* strains, which produce toxins that affect enzymes of plant arginine metabolism, causing devastating infections in crops. In this work, the structural and functional properties of plant N‐acetylornithine glutamate acetyltransferase (NAOGAT), the key deacetylating enzyme of the cyclic ornithine pathway and the target of mangotoxin, were investigated. We determined the structures of rice and maize NAOGATs to near‐atomic resolution. Plant NAOGATs demonstrate a characteristic ArgJ‐like N‐terminal nucleophile fold, undergoing autoproteolytic maturation into α and β subunits, generating an active‐site threonine nucleophile at the N‐terminus of the β‐chain. The enzyme catalyses acetyl transfer via a bi‐bi ping–pong mechanism in a bidirectional manner, although the acetylornithine‐to‐acetylglutamate conversion is preferred. NAOGAT undergoes minimal structural rearrangements during the reaction, however threonine acetylation causes pronounced changes in electrostatic potential around the active site which is also exhibited by the increased NAOGAT stability in the thermal shift assay. We did not detect clear evidence of NAOGAT acetylation by acetylCoA in our structural and biophysical data, which supports the specialisation of plant NAOGATs towards acetylornithine‐glutamate transacetylation. Finally, predictive bioinformatic analysis of the *P. syringae* mangotoxin‐producing operon, in the context of NAOGAT architecture, provided a basis for potential structural features of mangotoxin.

## Summary statement

- NAOGAT, key transacetylating enzyme in the cyclic ornithine pathway, is a target of mangotoxin, likely a dipeptide‐like inhibitor.
- Atomic resolution structures of rice and maize NAOGATs reveal the reaction mechanism with no support for acetyl‐CoA involvement, supporting monofunctionality of plant NAOGATs.

## 1 Introduction

Phytopathogens pose a great challenge to agricultural food production by causing substantial losses in crop yields. Looking at the invasion strategies of these pathogens provides insight into the specific metabolic pathways targeted by their infectious antimetabolites. Gaining an understanding of plant‐pathogen interactions at the molecular level opens many opportunities not only for managing plant diseases but also for identifying novel herbicide sites of action for weed control.

It is well established that arginine is the primary nitrogen storage molecule in plants (Winter et al. 2015; Yu et al. 2024). It is produced from ornithine in a pathway homologous to the urea cycle, involving the enzymes ornithine carbamoyltransferase (OTC), argininosuccinate synthase (ASS), and argininosuccinate lyase (ASL). Arginine can also be transformed back to ornithine by the action of arginase (ARGAH) with simultaneous urea release–the preferred form of nitrogen transportation in plants (Hildebrandt et al. 2015). Ornithine introduction into arginine metabolism is a crucial step in nitrogen assimilation. It enters the cycle via either a linear or cyclic pathway, catalysed by acetylornithine deacetylase (AOD, encoded by the *argE* gene, (Molesini et al. 2015, 2017)) or N‐acetylornithine glutamate acetyltransferase (NAOGAT, encoded by the *argJ* gene), respectively. Although NAOGAT is sometimes referred to in the literature as OAT (Cheng and Grishin 2005), OATase (Mishra et al. 2018), or OAT2 (Iqbal et al. 2011), the abbreviation NAOGAT is more descriptive and preferred here.

Among plant pathogens, *Pseudomonas syringae* pathovars are particularly noteworthy for producing antimetabolites that target many enzymes of nitrogen assimilation and arginine metabolism of the invaded plants (Arrebola et al. 2011a). The best‐characterised *P. syringae* toxins are tabtoxin and phaseolotoxin, which inhibit glutamine synthetase and OTC, respectively. According to Arrebola et al. (2003), another compound, mangotoxin, inhibits NAOGAT (and possibly AOD). Mangotoxin is described as a peptide with a molecular mass below 3 kDa. The *mbo* operon, directly responsible for mangotoxin production, is poorly characterised, and the exact chemical structure of mangotoxin remains elusive. It contains 6 protein‐coding genes (*mboA‐F*) (Carrión et al. 2013). Its regulation likely involves the two‐component gacS/gacA system, which influences regulatory *mgo* operon action (Carrión et al. 2014). Interestingly, *P. syringae* also harbours the *argJ* gene, suggesting that the bacterium needs to employ precautions against self‐inhibition of its own arginine metabolism. While defence against phaseolotoxin is achieved by possessing the second OTC isoform resistant to inhibition (Nielipinski et al. 2023), the mechanism preventing mangotoxin from inhibiting *P. syringae* NAOGAT remains to be elucidated.

The deacetylation step of N‐acetylornithine, disrupted by mangotoxin, is also not entirely understood in plants. Chemically, N‐acetylornithine serves as a precursor for ornithine in both linear and cyclic pathways, but the difference lies in the fate of the acetyl group. In the cyclic pathway catalysed by NAOGAT, the acetyl group is recycled by transfer to glutamate or possibly other amino acids. The reverse reaction is also possible, allowing ornithine to be converted into amino acids other than proline. Additionally, according to annotation (confirmed by SignalP), NAOGAT has an N‐terminal signal peptide that mediates its translocation to the chloroplast. It is believed that the cyclic pathway for ornithine production is characteristic not only for plants but also functions in non‐enteric bacteria and fungi (Molesini et al. 2015; Shargool et al. 1988; Slocum 2005). Some NAOGATs are described as capable of using acetyl coenzyme A (acetylCoA) as an acetyl group donor, facilitating the transformation of l‐glutamate to N‐acetylglutamate. Albeit, according to Marc et al. (2001), *Bacillus stearothermophilus* NAOGAT's affinity for glutamate is very low in the absence of acetylCoA.

NAOGATs are a part of the family ArgJ – annotated as bifunctional enzymes catalysing the first and fifth step in arginine biosynthesis, namely generation of acetylglutamate and generation of ornithine (Figure 1A). NAOGATs are also a part of the DmpA/OAT family, a functional N‐terminal nucleophiles (Ntn)–distinct from but sharing some features with the larger Ntn hydrolase superfamily (Cheng and Grishin 2005). Like all Ntn proteins, NAOGATs require autoproteolytic cleavage of the polypeptide chain to form α and β subunits. This chain break is required for enzymatic activity, as it creates the active site within a cleft between the two created subunits. In all enzymes of the Ntn hydrolase family, the catalytic residue is the newly formed N‐terminal residue of the β subunit. Hence the family name. This nucleophilic amino acid can be threonine, serine, or cysteine. The family also shares a common molecular assembly, namely a dimer of dimers (2α2β), once again ‐ a result of autoproteolytic action around the catalytic residue. The cleavage mechanism was described earlier and involves N → O(S) acyl shift mechanism (Buller et al. 2014; Devaraj and Perrin 2018), where O_ɣ_ of threonine performs a nucleophilic attack on C_α_ of the preceding residue. As a result, a 5‐element oxazolidine ring is created. It then breaks down, creating an Oɣ‐ester, which is ultimately hydrolysed to form α‐ and β‐subunits. Studies have shown (Marc et al. 2001) that mutation of the catalytic threonine to serine and cysteine slows autoproteolysis down, while mutating it to glycine absolutely abolishes the lysis. Unlike some Ntn‐hydrolases, NAOGATs do not undergo further proteolysis at the C‐terminus of the α‐subunit – none of the residues preceding the nucleophile residue are removed. NAOGATs are present in bacteria, fungi, and plants. The NAOGAT mechanism of action follows a bi‐bi ping pong kinetics, involving the formation of an acetylated intermediate (Figure 1B,C). The acetyl group is transferred from N_α_ of the amino acid donor (acetylornithine or acetylglutamate) to the O_ɣ_ of the N‐terminal threonine of the β‐subunit. This O‐acetylation likely proceeds via nucleophilic attack by the lone electron pair of Thr O_ɣ_ on the acetyl carbon with subsequent formation of the protein‐substrate intermediate complex, followed by the release of deacetylated amino acid and creation of the acetyl‐enzyme intermediate (Sankaranarayanan et al. 2010). Afterward, N‐acetylation of the second substrate is facilitated by nucleophilic attack of the lone pair electrons of its amino group. The amino group of the catalytic Thr is likely responsible for deprotonation of the substrate amino group, enhancing its nucleophilicity, which ensures postreaction regeneration of the enzyme (Duggleby et al. 1995; Linhorst and Lübke 2022). The reaction is reversible and can proceed in both directions, but the formation of acetylglutamate is highly favoured (see below).

**Figure 1:**
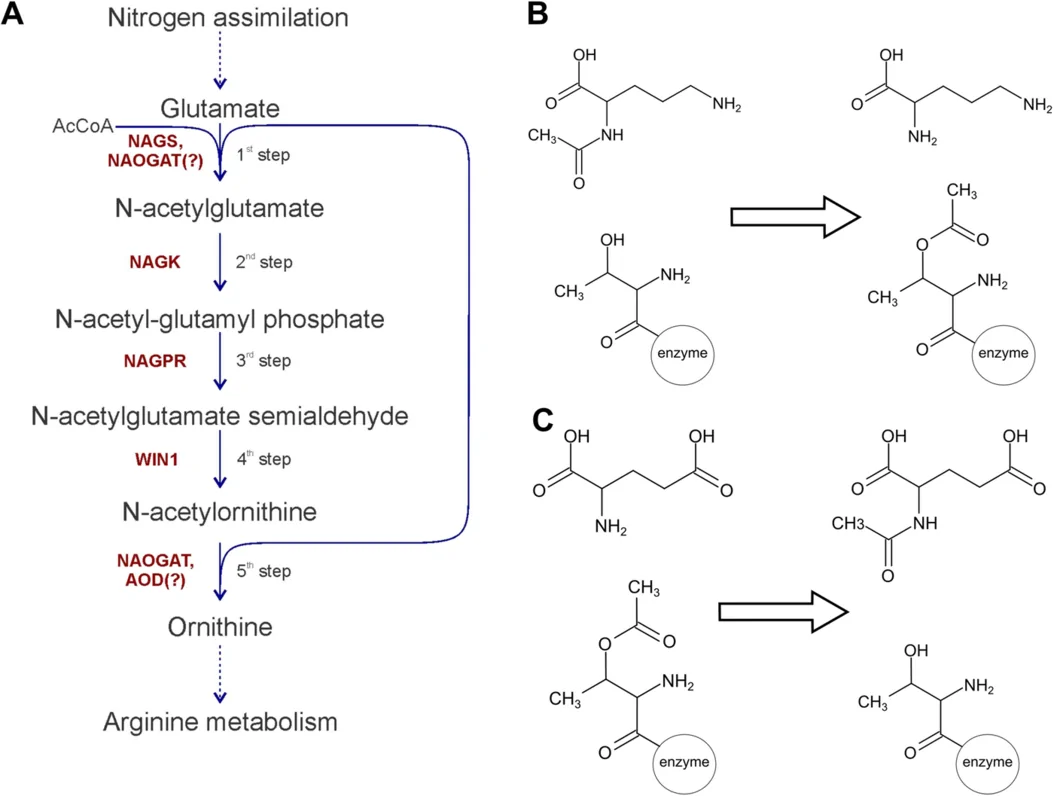
(A) Glutamate‐to‐ornithine conversion pathway in plants. Dotted arrows indicate transition from/to other parts of the pathway. Used enzyme abbreviations with UniProt IDs of *Arabidopsis thaliana* proteins: NAGS – N‐acetylglutamate synthase (Q84JF4), NAOGAT – N‐acetylornithine‐glutamate acetyltransferase (Q9ZUR7), NAGK – N‐acetylglutamate kinase (Q9SCL7), NAGPR‐ N‐acetylglutamyl‐phosphate reductase (Q93Z70), WIN1 ‐ acetylornithine aminotransferase (Q9M8M7), AOD – acetylornithine deacetylase (Q9C5C4). Question mark next to enzyme abbreviation indicates questioned enzymatic activity. (B) Schematic representation of acetyl intermediate formation during forward NAOGAT reaction and (C) recovery of unacetylated N‐terminal nucleophile (C).

Action of NAOGAT, and alternatively AOD, creates ornithine, an amino acid playing a pivotal role in plant development and mechanism of defence. During plant growth it serves as a precursor of proteinogenic amino acids – arginine and proline. It is also a substrate in polyamine production directly via ornithine decarboxylase (ODC) and indirectly through arginine decarboxylase (ADC) and arginase (ARGAH). The polyamines, in turn, take part in fruit set and flowering. Moreover, proline and polyamines play crucial roles for plant stress tolerance. The substrates of the NAOGAT reaction – acetylornithine and glutamate – are highly abundant metabolites and among the most affected under heat and drought stress conditions, with acetylornithine concentration rising nearly twenty‐fold (from 4.44 to 108.26 pmol mg^−1^ DW), and glutamate lowering by two‐fold (from 339.18 to 188.17 pmol mg^−1^ DW), in 7‐day trials on *Triticum aestivum* L. (Abdelhakim et al. 2022) However, plant stress response is more nuanced, and various metabolites, and their isoforms, are more prevalent under various conditions. The example of such metabolites are N‐α‐acetylornithine and N‐δ‐acetylornithine, excess of which is found in water‐stressed and well‐watered plants, respectively (Ćavar Zeljković et al. 2024). Additionally, N‐δ‐acetylornithine is believed to be a storage molecule for nitrogen, along arginine. Despite structural similarity between two isoforms of acetylornithine, the structural organisation of NAOGAT bindings pocket is consistent with preferential recognition of the N‐α‐acetylornithine isoform (see Results and Discussion section). The N‐δ‐acetylornithine synthesis is likely condition‐dependent and driven by l‐ornithine N5‐acetyltransferase (NATA1) via direct transfer of acetyl group from acetylCoA onto ornithine (Adio et al. 2011). On the other hand, N‐α‐acetylornithine synthesis is an integral part of arginine metabolism, controlled by N‐α‐acetylornithine aminotransferase protein (WIN1, also known as ACOAT or TUP5), and is crucial for plant development and root growth (Frémont et al. 2013).

According to InterPro (Blum et al. 2025), 36 ArgJ‐like protein structures, corresponding to 11 unique proteins, are known, but no plant homologue structures have been solved to date. This work reports the first structures of plant NAOGATs from rice (*Oryza sativa*) and maize (*Zea mays*) in their unliganded, acetylated and liganded forms. Both structural and biochemical evidence confirm the enzyme's function, direction of the reaction, and reversibility.

## 2 Results and Discussion

### 2.1 Plant NAOGATs General Structure

While initially we have successfully crystallised NAOGATs from 3 different plant species – *Arabidopsis thaliana, Oryza sativa* and *Zea mays*, structures of satisfactory quality were obtained only for *Os*NAOGAT and *Zm*NAOGAT. Although we have also determined the structure of *At*NAOGAT, it did not refine well, due to problems stemming from crystal lattice abnormalities. Despite screening through nearly 500 crystallisation conditions and using different additives, we could not overcome the issue. Hence, further structural analysis concerns *Os*NAOGAT and *Zm*NAOGAT structures, which were determined to near‐atomic resolution. *Os*NAOGAT and *Zm*NAOGAT contain signal peptides, spanning 39 and 33 residues, respectively. Thus, the prepared constructs did not contain signal peptides, making protomers 431‐ and 430‐residue long.

Both proteins present the same fold, and their biological assembly is a dimer of dimers – following conservative Ntn 2α2β tetrameric assembly (Figure 2A). A single αβ dimer is formed by cleavage of the polypeptide chain between alanine and threonine (see below) in a conserved ATML motif – residues 244–247 and 243–246 for *Zm*NAOGAT and *Os*NAOGAT, respectively. The cleavage most likely proceeds as described by Buller et al. (2014). The first chain (subunits α1 and α2) of the newly formed dimer contains 7 helices and 8 β‐strands. The other chain (subunits β1 and β2) contains 9 helices and 8 strands. Both autoproteolytically created subunits have a surface area of ~11 000 Å^2^, and they interface with each other with around ~2800 Å^2^. The interface between respective αβ dimers is smaller and comprises ~1500 Å^2^. The interface between subunit α and β is formed by residues 45–51, 60–68, 90–110, 118–130, 165–170, and 205–244 of the α‐subunit and 245–265, 273–300, 310–333 of the β‐subunit in *Zm*NAOGAT. In the *Os*NAGOAT situation is virtually identical, and the interface is created by the same structural elements. Single αβ dimer of NAOGAT comprises two structural domains – canonical and small (Figure 2A), which are not equivalent to post‐cleavage chains. The canonical domain comprises two β‐sheets flanked from left and right by helices, and by a helix from front and back; the structural elements are from both post‐cleavage subunits. Its fold is preserved throughout the whole ArgJ‐like group. The small domain comprises an antiparallel β‐sheet made of five strands and four helices, and those elements are only from the β‐subunit—this domain is not necessarily present in other ArgJ‐like proteins (see below).

**Figure 2:**
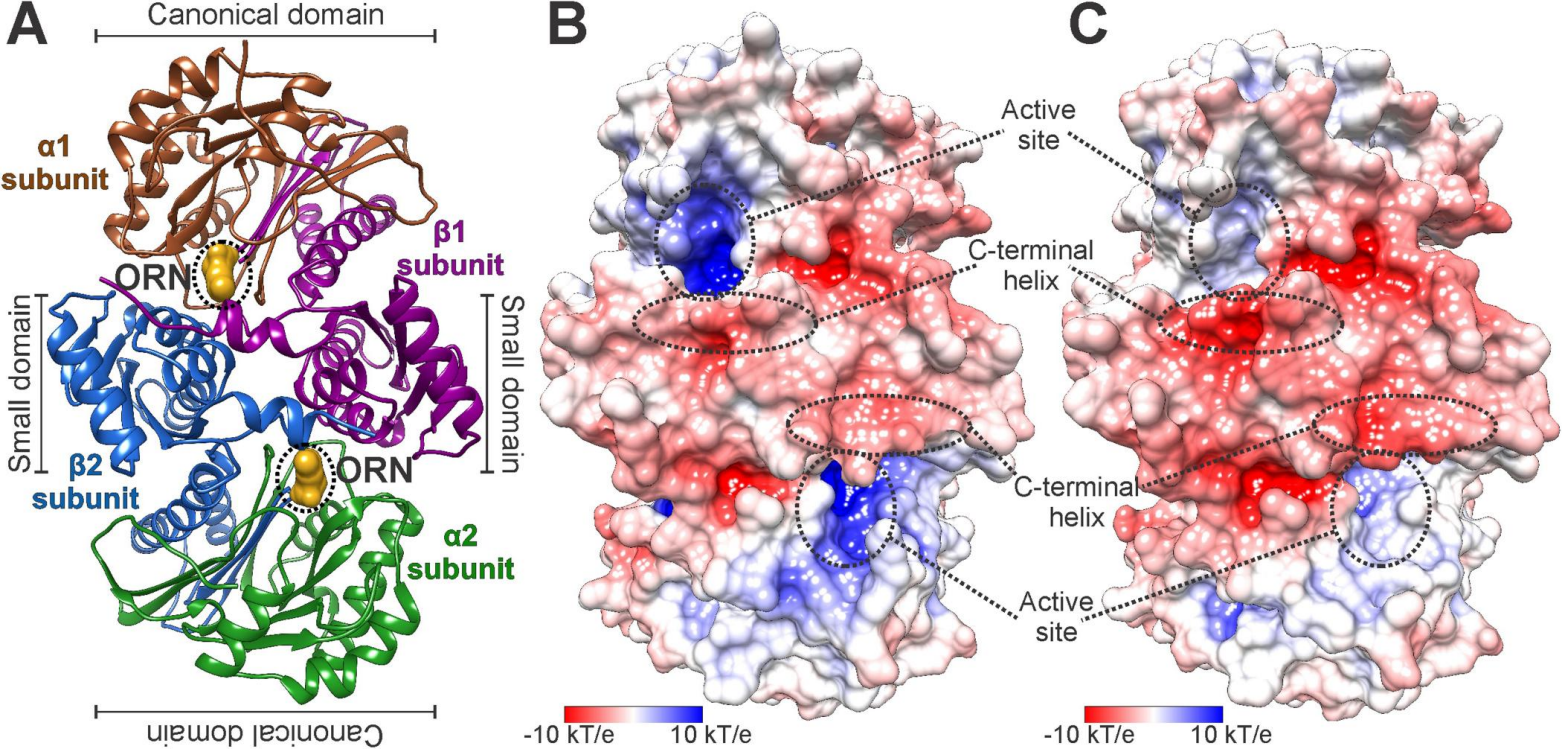
Structure of plant NAOGAT. (A) Ribbon representation of *Os*NAOGAT with bound ornithine (PDBID: 9TBN) presented as goldenrod surface. Distribution of surface electrostatic potential calculated at pH 7.5 of (B) nonacetylated *Zm*NAOGAT (PDB ID: 9TBP) and (C) acetylated *Zm*NAOGAT (PDB ID: 9TBO). [Color figure can be viewed at wileyonlinelibrary.com]

### 2.2 Active Site

The active site, formed by autocatalysis, is located in a cleft between a canonical and a small domain (Figure 2A). One of its walls is also formed by a small domain of the dimer mate. In both analysed NAOGATs, it has a volume of 700–800 Å^3^ with a surface area of 500–600 Å^2^ (depending on the analysed subunit). The most crucial element in the active site is the catalytic threonine of the aforementioned ATML motif – the newly created N‐terminal residue of the β‐subunit (chains B and D). An electrostatic potential calculation reveals that the active site of NAOGAT contains a big positively charged patch (Figure 2B), which is neutralised during acetylation of the catalytic Thr (Figure 2C). This is the binding site for ornithine in the *Zm*NAOGAT structure. Acetylation of the enzyme significantly improves its thermal stability (see below).

We have successfully obtained high‐resolution structures of *Os*‐ and *Zm*NAOGAT (acetylated and nonacetylated forms) with bound ornithine (Figure 3A,B) and the acetylated, ligand‐free *Zm*NAOGAT (Figure 3C). Acetylation of the rice protein is the result of crystallisation with acetylornithine. Our data show that ornithine, the product of the forward half‐reaction, stays bound in the active site regardless of the threonine acetylation (Figure 3A,B). O‐acetylation of *Zm*NAOGAT is the result of cocrystallization of the enzyme with acetylglutamate – structural evidence of a reverse half‐reaction (Figure 3C); however, glutamate dissociated from the active site and was not identified in the structure. The binding of the ornithine carboxylic moiety in nonacetylated *Zm*NAOGAT is facilitated by T_208_ and K_234_ from the α2 subunit and E_332_ from the β2 subunit; the sidechain amino group is stabilised by E_332_ and N_459_ of the β2 subunit. Ornithine α‐amino group is situated ~3 Å away from the catalytic threonine sidechain (Figure 3B). In *Os*NAOGAT, a similar ornithine binding mode is observed: carboxylic moiety is bound by T_207_ and K_233_ from the α2 subunit, and by E_331_ from the β2 subunit, and the sidechain δ‐amino group is bound by E_331_ and N_458_. The distance between the α‐amino group and catalytic threonine is also ~ 3 Å (Figure 3A). Superposition of both acetylated and nonacetylated structures of *Zm*NAOGATs reveals nearly no conformational changes within the active site. In place of the acetyl group at the catalytic threonine in the nonacetylated maize structure, a chlorine atom is bound in both protomers.

**Figure 3:**
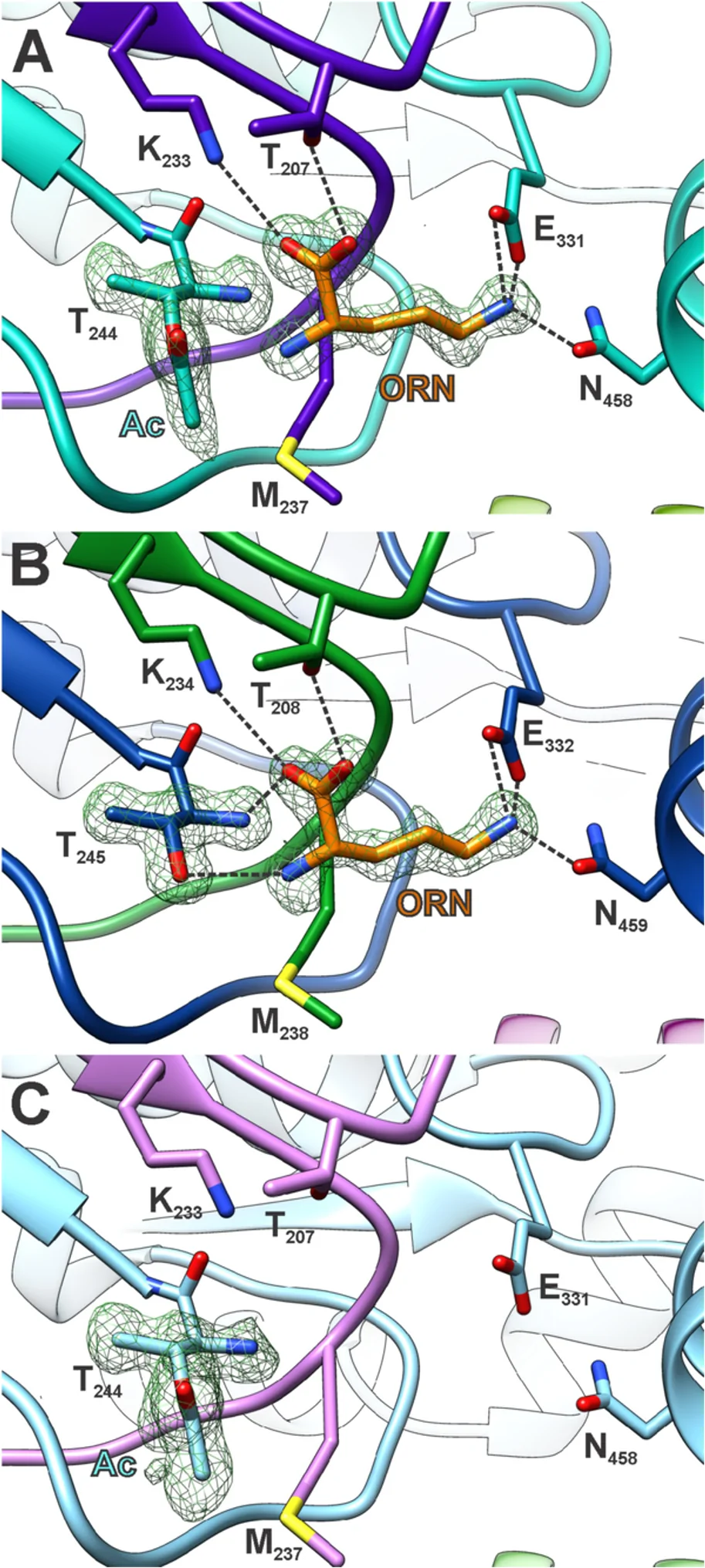
Active site of plant NAOGATs. (A) Ornithine (orange) bound within the active site of acetylated *Os*NAOGAT (teal and purple, PDBID: 9TBN); (B) ornithine bound within the active site of nonacetylated *Zm*NAOGAT (darkblue and green, PDB ID: 9TBP); (C) acetylation of catalytic threonine within the active site of *Zm*NAOGAT (cyan and violet, PDB ID: 9TBO). Crucial residues in the active site are depicted as sticks; dotted lines indicate interactions of the ligand with neighbouring residues; green mesh represents Polder omit maps contoured at 3σ around the catalytic threonine and bound ornithine. [Color figure can be viewed at wileyonlinelibrary.com]

Another interesting difference between acetylated and nonacetylated enzymes at the active site can be seen in surface electrostatic calculations (Figure 2B,C). It is apparent that acetylation of the active site threonine influences charge distribution on the surface of the protein. Acetylation balances charge inside the active site pocket. It also affects the negative potential of the C‐terminal helix (*Os/Zm*_452/453_YKYVEINAEYTT_463/464_), which is located near the catalytic site. This potential shift is reflected by the enzyme stability in the thermal shift assay and may regulate its activity. In other words, before the reaction, the unbalanced positive potential attracts N‐acetylornithine and ensures correct ligand positioning. After catalytic threonine is acetylated, ornithine's affinity for the active site decreases, allowing glutamate to replace ornithine in the active site. We have analysed structures of homological enzymes from *Mycobacterium tuberculosis* (*Mt*NAOGAT, PDB ID: 3IT4, 3IT6 (Sankaranarayanan et al. 2010) and *Streptomyces clavuligerus* (*Sc*NAOGAT, PDB ID: 2VZK (Iqbal et al. 2009) and noticed that C‐terminal residues following the C‐terminal helix protrude into the active site in structures if ornithine is bound (3IT6, Figure 4A) or the enzyme is acetylated (2VZK, Figure 4B). In the liganded *Mt*NAOGAT structure, it can be seen that the Nε of ornithine interacts with the side chain of the C‐terminal serine. Conformation of C‐terminal helix in *Os‐, Zm‐, Mt‐*, and *Sc*NAOGATs is virtually identical, and the only difference concerns the arrangement of the 5 terminal residues (EYTT for *Zm‐, Os‐*, and *Sc*NAOGAT, and AYSS for *Mt*NAOGAT). Distances between C_α_ of corresponding residues are ~2 Å for glutamate/alanine pair and from 4 to 5.8 Å for tyrosines (Figure 4A,B). Conformation of YTT residues in plant structures may be a subject to discussion due to the poor electron density map for the C‐terminus, which indicates their high mobility. This movement may be an effect of the aforementioned changes in electrostatic potential and a mechanism of substrate exchange and recognition. However, no movement of the helix was observed between acetylated and nonacetylated structures of *Zm*NAOGAT.

**Figure 4:**
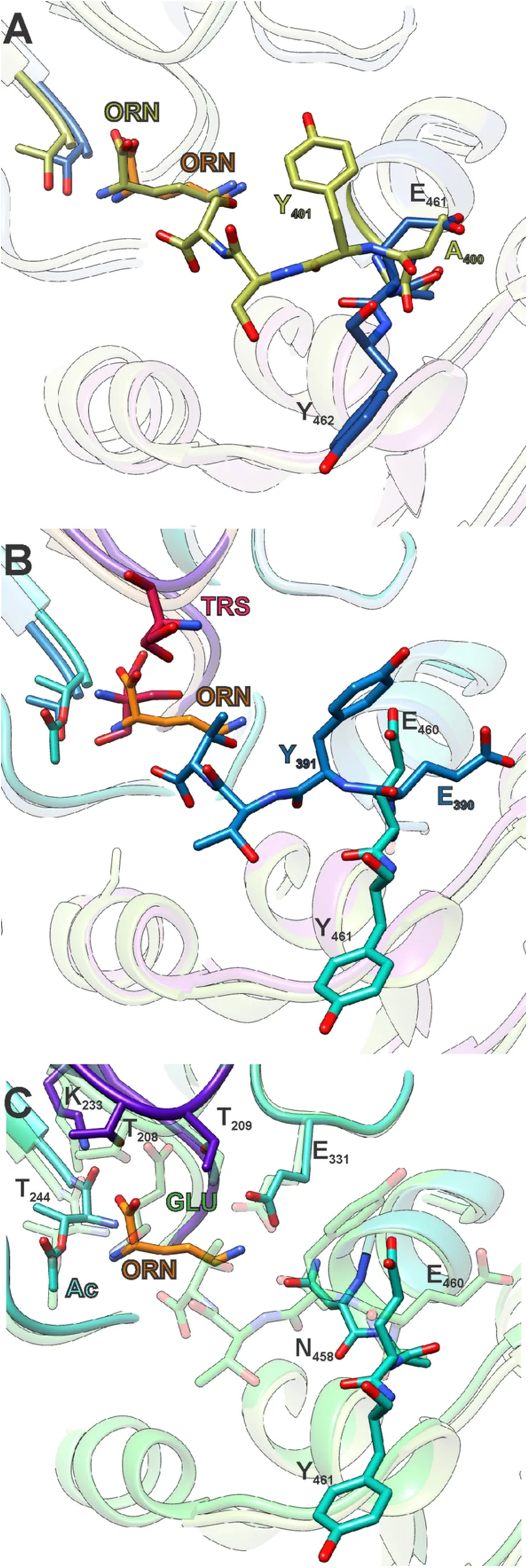
C‐terminal helix protrudes into NAOGAT active site. (A) Superposition of *Zm*NAOGAT (dark blue) with bound ornithine (ORN, orange) and *Mt*NAOGAT with ornithine (celery); (B) superposition of *Os*NAOGAT (teal) with ornithine (orange) and *Sc*NAOGAT (blue) with tris (TRS) bound (red); (C) superposition of *Os*NAOGAT (teal and purple) with bound ornithine (ORN, orange) with *Sc*NAOGAT (green) with glutamate (darkgreen). Some of the structural fragments are semitransparent for clarity; ligands and and key residues are depicted as sticks. [Color figure can be viewed at wileyonlinelibrary.com]

Even though both *Os*‐ and *Zm‐* NAOGATs were co‐crystallised with CoA and glutamate or acetylglutamate, we were not able to identify any of the ligands in electron density maps in any of the collected data. When NAOGATs were crystallised with acetylglutamate, electron density maps clearly indicated successful acetylation of N‐terminal threonine – the evidence for enzyme intermediate formation and reverse reaction catalysis (Figure 3C). However, the remaining part of the active site was empty, with no signs of bound glutamate. A structure of *Sc*NAOGAT with bound glutamate (PDB ID: 2YEP (Iqbal et al. 2011) is available. Superposition with our structures shows that the binding modes of the NAOGAT substrates (ornithine and glutamate) differ (Figure 4C). While the position of α‐amine groups is comparable, the sidechains of both amino acids adopt distinct conformations. Ornithine is pointing towards the C‐terminus of the β subunit, the one hypothesised to be responsible for substrate exchange and recognition. On the other hand, the glutamate sidechain is bound by T_148_ and T_149,_ in a cleft in the direct vicinity of the catalytic threonine. In *Os*NAOGAT, these residues are also threonines (T_207_ and T_208_), and they are responsible for the interaction with the carboxyl group of ornithine (Figure 4C). Iqbal et al. have shown that mutating any residue involved in glutamate binding reduces the enzyme activity by at least twofold. Interestingly, they also found that truncating the C‐terminal region lowers *Sc*NAOGAT activity by 2.5‐fold, which supports the importance of this region in substrate turnover. It is possible that the key difference between our crystallisation attempts, which prevented us from obtaining the complex with glutamate, is pH of the used buffers. *Os*NAOGAT was crystallised using a buffer with pH 6.5, and *Zm*NAOGAT crystals were obtained from the mother liquor at pH 5.0‐5.5. Meanwhile, Iqbal et al. (2011) have obtained their crystals using a buffer with a higher pH—7.5, closer to the theoretical pK value of amino group deprotonation (9.67), and further from the theoretical pK of sidechain deprotonation (4.25). Additionally, we have used 10 mM glutamate (a 20 × excess relative to the protein concentration), whereas they have used 100 mM.

#### 2.2.1 Plant NAOGAT Activity

We have tested substrate preference of *Os*NAOGAT and it is apparent that reaction with acetylornithine is 2.5 times faster than the reverse one (Supporting Information S1: Figure S1). Additional conducted test of using acetylmethionine, formylmethionine and acetylCoA as acetyl and general acyl group donor for ornithine have shown that NAOGATs are either unable to utilise those substrates or the reaction is extremely slow. We have crystallographically identified NAOGAT acetylation in structures incubated with both acetylornithine and acetylglutamate, but the enzymes remained deacetylated when it was incubated with acetylCoA, even in the long cocrystallization trials. Obtained near‐atomic resolution structural data clearly shows no electron density around catalytic threonine in the structure obtained from crystal incubated with acetylCoA. Even 2 h‐long preincubation of NAOGAT with 100× molar excess of acetylCoA did not cause statistically significant increase of the enzyme thermal stability in the SYPRO orange assay (see below), showing no pronounced acetylation or high‐affinity binding. Marc et al. 2000, have investigated NAOGATs from thermophilic organisms *Bacillus stearothermophilus*, *Thermothoga neapolitana* and an archea *Methanococcus jannaschii*. NAOGATs displaying dual activity (*B. sterothermophilus* and *T. neapolitana*) were 5‐10 times less active in presence of acetylCoA and glutamate than with acetylornithine and glutamate. *Bs‐* and *Tn*NAOGATs activity was supported by the detection of acetyl intermediate formation using radiolabeling studies with actetylCoA. On the other hand, *M. jannaschii* NAOGAT showed no detectable dual activity and was not modified with radiolabelled acetylCoA. That leaves the question if the plant NAOGATs are really capable of serving the dual role, or are they monofunctional, like *M. jannaschii* NAOGAT. There are also other examples of monofunctional NAOGATs from *Corynebacterium glutamicum* (Sakanyan et al. 1996) and *Thermus thermophilus* (Baetens et al. 1998). They share ~36% and 44% sequence identity with both *Os*‐ and *Zm*NAOGATs. The sequence analysis of NAOGATs (see below) situated plant, *T. thermophilus* and *M. jannaschii* (both monofunctional) proteins in the same clade and *C. glutamicum* in different clade. Errey and Blanchard (2005) theorised that bifunctionality of NAOGAT is connected to absence of either *argA* gene encoding N‐acetylglutamate synthase (NAGS) protein or other genes encoding protein with similar activity. In case of a model plant *A. thaliana* and the host plants of analysed proteins *O. sativa* and *Z. mays* at least two, three and five putative NAGS can be found in their genomes, although the definitive function of these proteins was never established.

To further investigate interactions of plant NAOGAT with substrates (acetylornithine, acetylglutamate) and products of half‐reactions, as well as acetylCoA and CoA, we have performed differential scanning fluorimetry (DSF, Figure 5A,B) assay with SYPRO orange that measures thermal shift of protein's apparent melting temperature (T_m_). The protein alone displayed T_m_ of 59.63 ± 0.95°C and 57.00 ± 0.5°C with 10% DMSO (Figure 5C and Supporting Information S1: Table 1). We have tested T_m_ of *Zm*NAOGAT using three different ligand concentrations (protein:ligand molar ratio 1:4, 1:20, 1:100). After 2 h of incubation, we have observed statistically significant change of T_m_ for treatments with 1 and 5 mM acetylornithine and acetylglutamate (Figure 5C). Additionally, the observed effect for 5 mM ornithine was also statistically significant. The T_m_ increased up to 67.25 ± 0.50°C and 71.13 ± 0.25°C for the highest substrate concentration, for acetylornithine and acetylglutamate, respectively. For other tested variants the shifts were statistically insignificant (Figure 5C.). Given the fact that glutamate and ornithine presence does not facilitate NAOGAT stability as preincubation of the protein with acetylglutamate and acetylornithine, the increase of T_m_ by over 7 and 11 degrees arise from the catalytic threonine acetylation. We did not observe pronounced stabilising interactions with acetylCoA and CoA; no significant increase in T_m_ with 100× molar excess of acetylCoA within 2 h of incubation was observed. These observations are in line with our crystallographic data – no detected acetylation of NAOGAT by acetylCoA or cofactor binding in our structural studies. The results support monofunctionality of plant NAOGATs, like in *M. jannaschii, T. thermophilus*, or *C. glutamicum*. The specialisation of NAOGAT is especially important for the plant stress response. According to Abdelhakim et al. (2022), both acetylornithine and glutamate are among the most accumulated in plant tissues during prolonged heat and drought stress in *Triticum aestivum* L. Interestingly, analysis of differential gene expression (GSE217631) of *Solanum lycopersicum* under salt stress shows that *Sl*NAOGAT (Solyc09g010420.3) was not found to be differentially expressed under stress condition. At the same time, expression of acetylornithine aminotransferase (Solyc08g080370.3), the enzyme responsible for the catalysis of the preceding reaction in the cycle, rose significantly in tomato roots under the salt stress (Ikuyinminu et al. 2023). The results suggest that the elevated levels of NAOGAT substrates are more likely connected with upregulation of other enzymes in the pathway rather than alteration of NAOGAT expression or activity.

**Figure 5:**
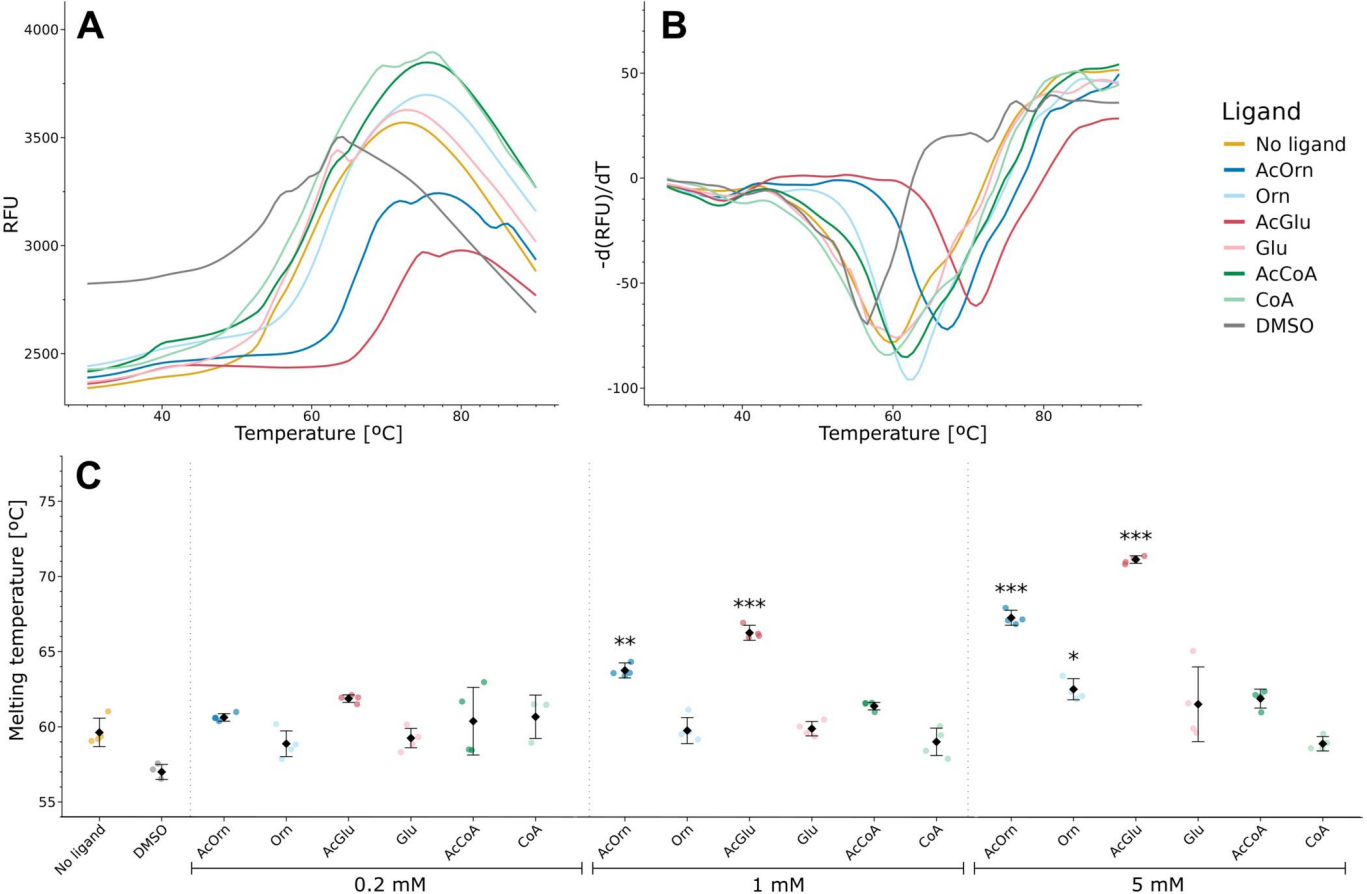
Results of thermal shift analysis on *Zm*NAOGAT, acetylated derivatives are depicted as darker and non‐acetylated ligands are in lighter colour – blue for ornithine, red for glutamate, green for CoA. Non‐modified protein is depicted as golden, and protein in 10% DMSO is in grey. (A) Melting curves of protein without ligand, with 10% DMSO and in the presence of 5 mM of various ligands; each curve is depicted in different colour and corresponds to the ligand used in the assay (see legend on the right). (B) First derivative of measured fluorescence plotted against temperature. (C) Comparison of melting temperatures (Tm) across analysed ligand concentrations. Black diamond depicts averaged Tm values from four replicates (coloured dots), with marked standard deviations; asterisks denote statistically significant different Tm as compared to unliganded protein. Significance was calculated using type II ANOVA on joined groups with Games‐Howell post‐hoc test (*p < 0.05, **p < 0.01, ***p < 0.001). [Color figure can be viewed at wileyonlinelibrary.com]

Initially we suspected that the deciding aspect of our crystallisation attempts, which prevented us from obtaining the complex with glutamate, is pH of the used buffers. *Os*NAOGAT was crystallised using a buffer with pH 6.5, and *Zm*NAOGAT crystals were obtained from the mother liquor at pH 5.0–5.5. Meanwhile, Iqbal et al. (2011) have obtained their crystals using a buffer with a higher pH (7.5), which is closer to the theoretical pK value of amino group deprotonation (9.67), and further from the theoretical pK of sidechain deprotonation (4.25). Additionally, we have used 10 mM glutamate (a 20 × excess relative to the protein concentration), whereas they have used 100 mM. Although our thermal shift analysis mimicked these conditions, with a buffer at pH 7.8 and even higher ligand excess, we did not observe statistically significant change in T_m_, which indicates that native *Zm*NAOGAT likely does not create stable complex with glutamate.

### 2.3 Comparative Analysis of ArgJ‐Like and Ntn Hydrolases

At first glance, both ArgJ‐like (with NAOGATs) and Ntn hydrolases appear very similar. In the past, they were even wrongly classified as a part of a single superfamily. According to Cheng and Grishin (2005) ArgJ‐like and Ntn hydrolases are products of divergent evolution, rather than evolving from a common ancestor. The similar features are visible in both groups sharing a comparable fold, requiring autoproteolysis for activity, and relying on similar molecular mechanism of action. At the same time, both groups are rather diverse, with their member proteins displaying a plethora of enzymatic activities. Here we present further structural evidence supporting separation of ArgJ‐like domain‐containing proteins from Ntn hydrolases.

To elucidate similarities and differences of both NAOGATs and Ntn hydrolases we have analysed 451 protein sequences belonging to both families. Out of these, 308 had UniProt annotation of EC number. All annotated proteins similar to *Os*NAOGAT identified through BlastP exhibit either transferase or dual transferase activity – these are NAOGATs. In case of Ntn hydrolases family, the range of activities is more diverse. Additionally, proteins belonging to this superfamily are annotated as mono‐, bi‐ or tri‐ functional hydrolases, transferases or hybrid transferases/hydrolases; there are also ligases or oxidoreductases. Regarding the residue acting as the N‐terminal nucleophile, the Uniprot database provides this information for 316 out of the 451 analysed sequences. Only one protein belonging to *ArgJ* family, namely a ArgJ2 protein from *Streptomyces clavuligerus* (UniProt ID: P62252), putatively uses an N‐terminal serine, while all other NAOGATs use threonine. Similarly, in Ntn hydrolases the role of an N‐terminal nucleophile is performed mostly by threonine, although some proteins are functional with serine or cysteine performing this role. In carbapenem‐3‐carboxylate synthase from *Pectobacterium carotovorum* subsp. *carotovorum* (PDB ID: 1Q15 and 1Q19 (Miller et al. 2003), UniProt ID: Q9XB61) the active residue is putatively tyrosine. Analysis of these structures indicate that neither putatively active tyrosine is N‐terminal in β‐subunit, nor has autoproteolysis occurred; likely this protein displays similar fold to Ntn hydrolase superfamily, yet does not belong to it.

The feature which links proteins displaying plethora of different activities is a shared fold that enables autoproteolytic activation and formation of a catalytic pocket as the result of proteolysis. ArgJ‐like domain superfamily (IPR016117) is represented by 36 structures in the PDB. Among them, there are 11 unique protein structures from different organisms and only 3, which correspond to bacterial NAOGATs – *Mycobacterium tuberculosis, Streptomyces clavuligerus*, and *Halalkalibacterium halodurans*. Other structures sharing the ArgJ‐like fold are: beta‐peptidyl aminopeptidase (BapA, PDB ID: 3NFB (Merz et al. 2011), D‐aminopeptidases (DmpA, PDB ID: 1B65 (Bompard‐Gilles et al. 2000), 6‐aminohexanoate‐oligomer (nylon) endohydrolase (NylC, PDB ID: 3AXG (Negoro et al. 2012) and pantheteine hydrolase (ThnT, PDB ID: 3TM1 (Buller et al. 2012). On the other hand, Ntn hydrolase superfamily members which share similar mechanism to NAOGATs, are: glutamate synthase (*α*GltS, PDB ID: 1EA0 (Binda et al. 2000), ferrodoxin‐dependent glutamate synthase (GltS, PDB ID: 1LLW), glutathione hydrolase (GGT, PDB ID: 2DBU (Okada et al. 2006), amidophosphoribosyltransferase (PRPP, PDB ID: 1AO0 (Chen et al. 1997), glutamine‐fructose‐6‐phosphate aminotransferase (GFPT1, PDB ID: 2V4M (Moche et al. 2008), β‐subunit of proteasome (threonine endopeptidase, Psmb3, PDB ID: 3UNB (Huber et al. 2012) and isoaspartyl peptidase (*Ec*AIII, PDB ID: 1JN9 (Michalska et al. 2008).

While not all structural elements are present in both NAOGAT and ThnT proteins, they follow the same general fold, with central β‐sheet flanked by helices. The main difference between the two is that ThnT lacks several residues from the C‐terminus of the α subunit, which are autoproteolytically removed during the chain cleavage, and the whole domain corresponding to NAOGAT's C‐terminal domain (also referred as small, Figure 6A). Superposition of the two structures reveals that central sheet conformation is virtually identical, with N‐termini of the newly created β subunits superposing well in the same place. On the other hand, direct comparison of *Os*NAOGAT with exemplary Ntn‐hydrolase isoaspartyl peptidase (asparaginase) from *Phaseolus vulgaris* (PDB ID: 4PU6 (Bejger et al. 2014) shows that while the general observable fold of αββα sandwich is preserved and, in both cases, autoproteolytic action has occurred revealing N‐terminal nucleophile residue in the binding site the spatial orientation of the secondary structure elements is significantly different. Superposition of *Os*NAOGAT and *P. vulgaris* asparaginase structures reveal that position of the central sheet differs massively in both proteins (Figure 6B). While similar at the first glance, they are very distinguishable. All of that confirms that while both NAOGATs and Ntn hydrolases rely on similar mechanisms, they stem from different evolutionary background and hence are not easily comparable.

**Figure 6:**
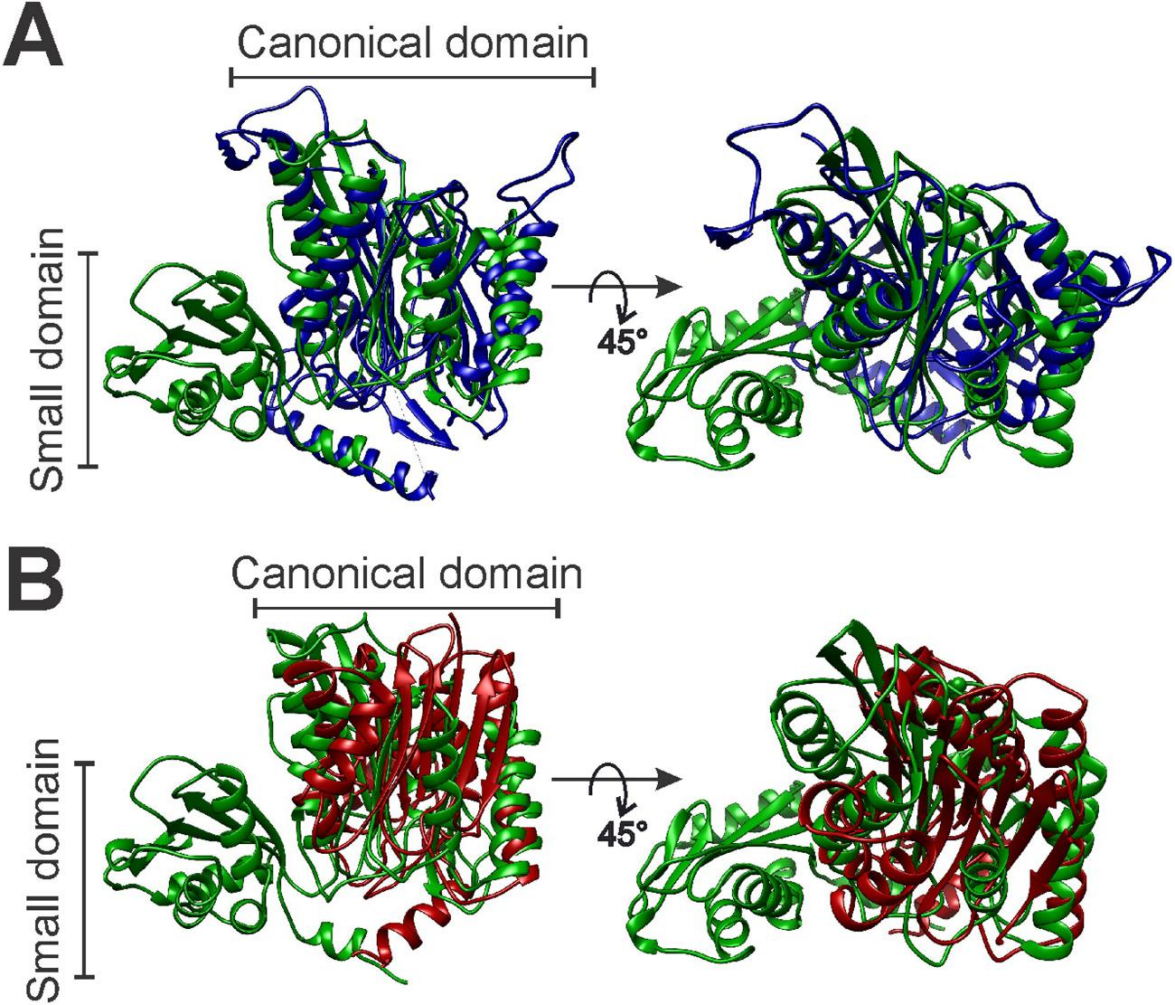
Comparison of NAOGATs and Ntn hydrolases. Superposition of post‐cleavage heterodimers of *Os*NAOGAT and (A) pantetheine hydrolase from *Streptantibioticus cattleyicolor* (PDB ID: 3TM1 (Buller et al. 2012), B) plant‐type L‐asparaginase from *Phaseolus vulgaris* (PDB ID: 4PU6 (Bejger et al. 2014). [Color figure can be viewed at wileyonlinelibrary.com]

### 2.4 Sequence Analysis of NAOGATs

*Os*‐ and *Zm*NAOGAT share 87% of sequence identity – most of the differences being found between their transit peptides. When compared without them the identity jumps to 91%. In comparison NAOGAT from the model plant species *Arabidopsis thaliana*, devoid of transit peptide, shares ~73% of sequence identity with both analysed structures.

Usage of blastP (Camacho et al. 2009) against SwissProt (Famiglietti et al. 2019) database reveals 204 sequence homologues with sequence identity varying from 25% to 100%, belonging to either bacteria, fungi or plants. ArgJ analysis using XSTREME (Bailey et al. 2015; Grant and Bailey 2021) identified 12‐residue long motif preceding and including proteolytic site (Figure 7A) in almost all proteins. The analysis highlighted another characteristic motif, following the cleavage site (Figure 7B). It exhibits higher variability but still was found in 197 of the analysed proteins. The variable motif contains a conserved cluster (TDA) exhibiting above‐average conservation compared to other residues in that motif (residues 251–253 and 252–254 in *Os*‐ and *Zm*NAOGATs, respectively). Those residues are not in an immediate vicinity of the active site, neither they bind any ligands or ions. They likely exhibit an important structural function – are responsible for bending of protein chain and connecting strand containing catalytic threonine with the following helix securing the correct fold of the protein.

**Figure 7:**
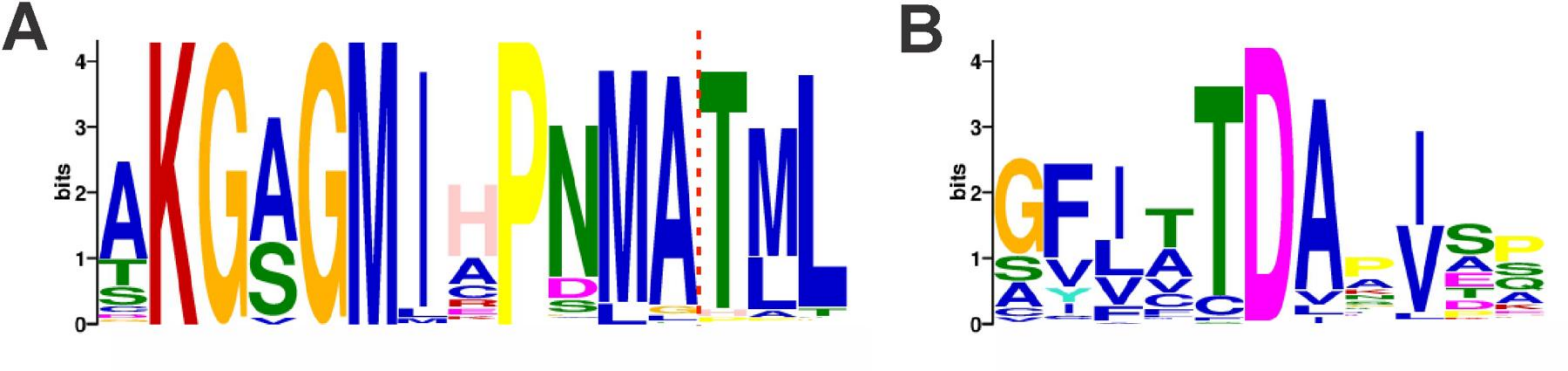
Characteristic motifs found in NAOGATs. WebLogo representation of the motifs found with XSTREME analysis: (A) MATL motif preceding and containing cleavage site, (B) TDA motif responsible for loop bending; Vertical red dotted line shows the autoproteolytic cleavage site; Each sequence position is presented as stacks of symbols, where the overall height of the stack indicates the sequence conservation at that position, while the height of symbols within the stack indicates the relative frequency of each amino acid at that position. [Color figure can be viewed at wileyonlinelibrary.com]

After aligning sequences of both NAOGATs and Ntns and constructing a phylogenetic tree (Figure 8), it can be seen that they group into separate clades. Additionally, while NAOGATs are clustered together by taxonomy, Ntn hydrolases appear more mixed. It is due to the aforementioned fact that Ntn hydrolase family is very diverse and the difference in function manifests itself in difference in sequence, hence, proteins cluster by function rather than taxonomy. Additionally, in NAOGATs only one protein (*Streptomyces clavuligerus* NAOGAT2, UniProt ID: P62252) is marked as having different nucleophile residue – serine instead of threonine (Figure 8).

**Figure 8:**
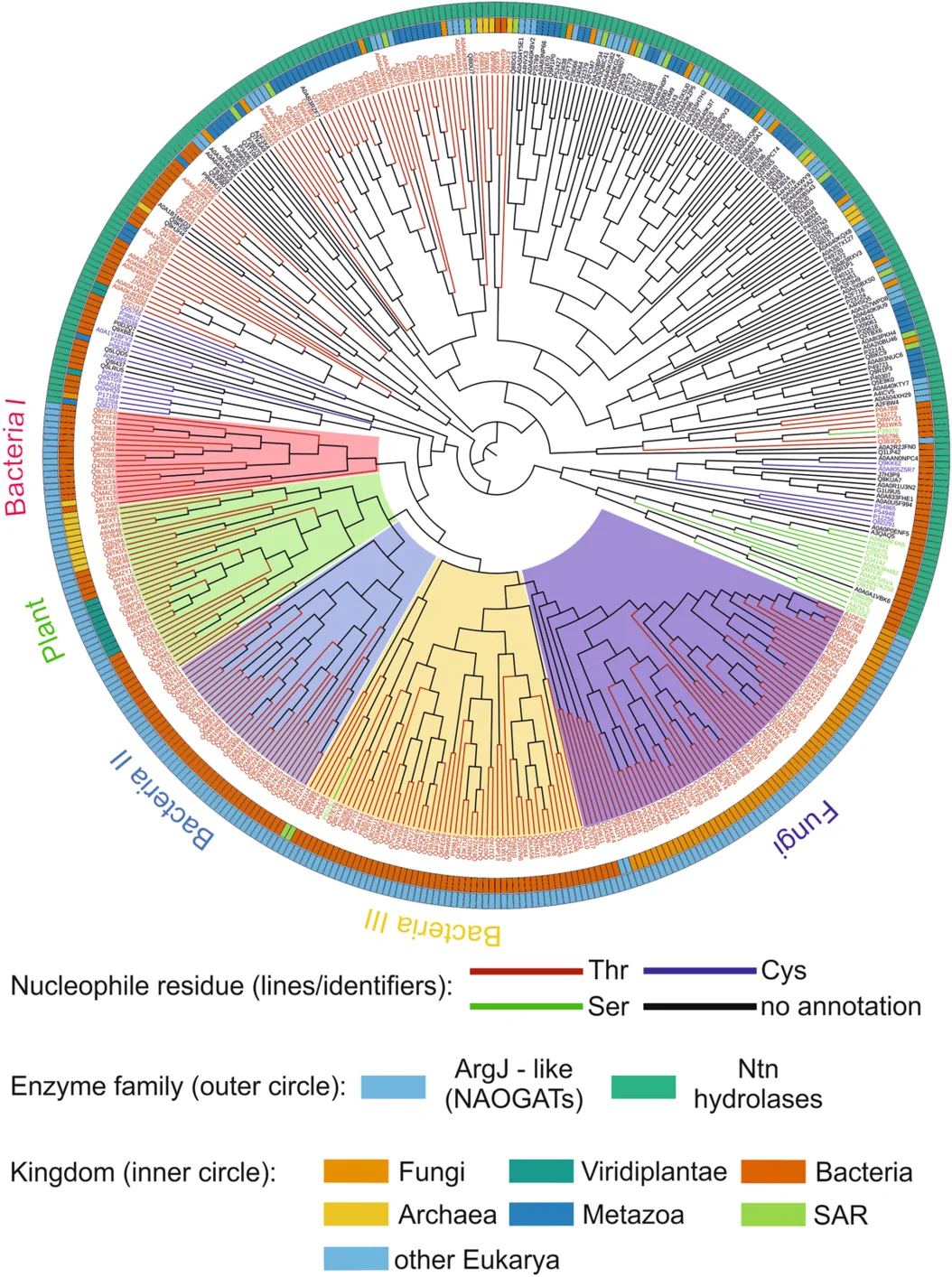
Phylogenetic tree of ArgJ and Ntn‐hydrolase proteins. Edge and name colour describe annotated nucleophile residue: red – threonine, blue – cysteine, green – serine and black – no annotation, wedges in inner circle describe protein host organism affiliation to phylogenetic group/clade light orange – Fungi, green – Viridiplantae, dark orange ‐ Bacteria, yellow – Archaea, blue – Metazoa, light green – SAR, light blue – other Eukarya, outer circle wedges describe affiliation of protein to ArgJ‐like family (light blue) or Ntn‐hydrolases (green). [Color figure can be viewed at wileyonlinelibrary.com]

Closer look at the part of the tree with NAOGATs reveals five distinct clades designated as Bacteria I–III, Plant and Fungi (Figure 8). Bacteria I clade contains 18 sequences – 17 of which are bacterial. The only outlier is archaeal NAOGAT from *Methanopyrus kandleri* (UniProt ID: Q8TX15). The Plant clade is the most diverse taxonomically out of analysed sequences. It contains archaeal, plant and bacterial proteins. Bacterial sequences in this clade are derived either from extremophile organisms like *Thermus thermophilus* or *Deinococcus radiodurans*, cyanobacteria – *Nostoc, Synechococcus, Synechocystis, Procholoroccus* and others. As mentioned before NAOGAT is a chloroplastic protein and the similarity between plastid proteins and cyanobacterial is understandable in a light of theory of endosymbiosis (McFadden 2014; Sato 2021). Similarly, it is believed that oxygenic photosynthesis is of archaean origin, hence the archaeal proteins in the clade (Fournier et al. 2021; Lei and Burton 2021). Both Bacteria II and Bacteria III clades are mostly composed of bacterial protein with only two exceptions of the SAR clade – proteins from *Phytophtothora infestans* and *Thalassiosira pseudonana*. When it comes to Fungi clade it mostly comprises fungal sequences with exceptions being proteins from *Heterostelium pallidum* and *Dictyostelium discoideum* – both also belonging to eukaryotes, and few bacterial sequences.

### 2.5 Plant NAOGATs as *Pseudomonas syringae* Targets

Mango trees are prone to infection of *Pseudomonas syringae*. These bacteria pathovars can produce various toxins blocking activity of different enzymes of plant nitrogen metabolism – tabtoxin blocking glutamine synthase, phaseolotoxin blocking OTCs and finally mangotoxin blocking NAOGATs (Arrebola et al. 2011b). Both, tabtoxin and phaseolotoxin are well characterised with their molecular structures elucidated. In case of mangotoxin, only known things are that it is most likely a peptide‐like molecule with mass lower than 3 kDa and that it is produced as an action of *mbo* and *mgo* operons. It was established by mutation and complementation studies that insertion mutation of most of the genes in these operons results in loss of ability to produce mangotoxin (Arrebola et al. 2003, 2007, 2009, 2012; Carrión et al. 2012). Probable connection between the two operons is that *mgo* operon is controlled by gacS/gacA system and is responsible for production of signalling molecule which kickstarts the *mbo* operon and production of mangotoxin (Arrebola et al. 2012; Gutiérrez‐Barranquero et al. 2019). We have decided to update assignment of putative roles and ligands of *mbo* and *mgo* operon proteins (Arrebola et al. 2007) leveraging newest databases (Famiglietti et al. 2019; Blum et al. 2025; Sayers et al. 2025) and software (Supporting Table 2).

The *mgo* operon is the smaller of the two analysed operons and probably contains 4 proteins: mgoA‐D. Arrebola et al. (2012) have proven that all of *mgo* genes are co‐expressed under a single promoter and as a single transcript. Upstream of the *mgo* operon a gene coding regulatory protein belonging to GntR family (UniProt ID: Q19AK4, PDB ID: 3C7J) (Nocek et al. 2008) is found. The regulator contains HTH DNA binding domain at N‐terminus and an oligomerization/ligand binding domain at C‐terminus. However, the function or connection of this regulatory protein to the mangotoxin production is unknown. Composition of the *mbo* operon likely confirms that mangotoxin is a peptidic compound (mboC and mboD are both ligases, likely forming an amide bond), probably, but not necessarily, containing a ring moiety (mboB). Mutational analysis conducted by Carrión et al. (2012) shows that mutants with defective either *mboE* or *mboF* genes were still capable of producing mangotoxin. However, without knowledge on the structure of mangotoxin it is very hard to infer exact functions of the proteins in *mbo* operon or even the order of their action. The only information found regarding the toxin structure is that it is probably “composed of two amino acids linked to sugar” (Arrebola et al. 2011a).

Another unanswered question is why *P. syringae* is able to produce a toxin blocking NAOGAT while surviving on its own. In the case of OTC, *P. syringae* pathovars producing phaseolotoxin have two OTCs in their genomes, one susceptible to the toxin on the other resistant to it (Nielipinski et al. 2023; Peet and Panopoulos 1987). Some strains of *Streptomyces clavuligerus* have multiple NAOGAT versions encoded in their genomes (Tahlan et al. 2004). Additionally, those proteins display only ~30%–40% sequence similarity between each other, and two of them are likely involved in clavulonic acid synthesis and only one is a housekeeping urea cycle enzyme. *Ps*NAOGAT shares ~35% sequence similarity with either *At*‐, *Os‐* and *Zm*NAOGATs. No structure of *Ps*NAOGAT was experimentally solved. The closest homologue of this protein with almost full sequence coverage is NAOGAT from *S. clavuligerus* (PDB ID: 1VZ6, 1VZ8 (Elkins et al. 2005) which shares 34% of sequence similarity. Model of *Ps*NAOGAT predicted with AlphaFold3 using AlphaFold server (Abramson et al. 2024) shows that with pLDDT over 95 it shares characteristic fold and all secondary structure elements with other NAOGATs.

In order to check if toxin resistance mechanism is similar to the OTC ‐ a paralogous protein with similar fold but dissimilar sequence, Foldseek (van Kempen et al. 2024) was used to scan Alphafold (Jumper et al. 2021) predictions of *Pseudomonas* genus and *P. syringae* proteomes. Only single copy of NAOGAT protein was found in proteomes of any *Pseudomonas* genus bacteria. It is possible that defence against the compound is not needed, as it requires further processing to become active. It is also possible that the further processing is done only *in planta* – similarly to what happens with phaseolotoxin; however, in contrary to phaseolotoxin, the mangotoxin precursor may be not active, hence there would be no need for additional NAOGAT enzyme in *P. syringae*. This notion is seemingly undermined by other experiments, as Arrebola et al. (2003) have shown that *P. syringae* is capable of inhibiting growth of *E. coli* through mangotoxin production. Since *E. coli* lacks *argJ* gene coding NAOGAT, and the inhibitory effect was reversed by ornithine and not by acetylornithine, mangotoxin is suspected to also block AOD activity.

Without structure of mangotoxin it is hard to establish its inhibition mechanism, but it is possible to infer if mangotoxin is bigger or smaller than phaseolotoxin or tabtoxin by analysing proteins inhibited by the other toxins and their binding pockets, assuming that all of the toxins are competitive inhibitors binding in the active site rather than allosteric ones. We have performed the analysis with kvfinder (Oliveira et al. 2014) in ChimeraX (Meng et al. 2023) using the structures of glutamine synthetase (GS) from *Zea mays* (PDB ID: 2D3C (Unno et al. 2006), ornithine carbamoyltransferase (PDB ID: 8QEU (Nielipinski et al. 2023), ornithine decarboxylase from *Trypanosoma brucei* (PDB ID: 1QU4 (Grishin et al. 1999), a protein with solved structure exhibiting the highest sequence similarity to plant ODC), and *Escherichia coli* AOD (PDB ID: 7RSF (Osipiuk et al. 2021). Chosen inhibitory compounds are antimetabolite toxins produced by *P. syringae* and p‐chloromercuribenzoic acid, a commercial protease inhibitor also described as the NAOGAT inhibitor (Staub and Dénes 1966). It is worth noting that we have failed to crystallise NAOGAT complex with p‐chloromercuri benzoic acid due to severe protein precipitation in contact with the ligand. The effect of protein denaturation may be worth considering when analysing inhibitory effect of p‐chloromercuri benzoic acid on NAOGATs.

The analysis of binding sites and ligands shows that the ratio between approximate volumes of a ligand and a binding site differ between 1:2 and 1:4 (Supporting Table 3). This, while analysed on much smaller scale, coincides with the findings of Saranya and Selvaraj (2009) where they have established that the aforementioned ratio varies from 1:2 to 1:5 and is rather stable for specific enzyme family. Both enzymes recognised as inhibited by mangotoxin – a plant *Zm*NAOGAT and *Ec*AOD have comparable binding sites volumes. Based on that fact and assuming that mangotoxin like other toxins produced by *P. syringae* is a competitive inhibitor rather than allosteric, we suspect that the volume range for mangotoxin is ~250–300 Å^3^ – a volume of dipeptide with aromatic ring, i.e. Tyr‐Glu (Supporting Table 4). This volume would correspond to a compound with a mass of 220‐330 Da if its composition is similar to other analysed compounds (Supporting Table 5). Additionally, *Zm*NAOGATs binding site is positively charged, as established by APBS (Jurrus et al. 2018) calculation, and if the mangotoxin is bound in the active site it most possibly possesses electron donor characteristic. In summary, the predictive analysis of *P. syringae* mangotoxin‐producing operon confronted with NAOGAT architecture has led to the hypothesis that mangotoxin could be a small, negatively charged dipeptide‐like inhibitor that may act as a competitive ligand within the active site. However, this assumption could only be confirmed, when the mangotoxin is isolated and characterised structurally.

## 3 Conclusions

We have obtained first crystal structures of plant NAOGATs in both their acetylated and nonacetylated forms with their biological ligand (ornithine) bound. Various procedures and conditions used to grow the crystals allowed us to confirm transacetylation activity of the enzymes in forward and reverse half‐reactions. Activity of the plant NAOGATs was also supported by the enzymatic assays in both directions, although activity towards acetylornithine was much higher in comparison to acetylglutamate. Thermals shift assays, showing increased stability of plant NAOGAT after incubation with the acetylated substrates are in line with enzymatic studies. At the same time, we were unable to identify enzyme acetylation in the structures derived from crystals incubated with acetylCoA. We also did not observe increased enzyme stability with acetylCoA in DSF which would indicate enzyme acetylation in half‐reaction. These findings suggest that plant NAOGATs are specialised for acetylornithine‐glutamate transacetylation, similarly to paralogous enzymes from *M. jannaschii, T. thermophilus*, or *C. glutamicum*. Based on the performed phylogenetic analysis, which clearly differentiates Ntn‐hydrolases and ArgJ‐like enzymes, we have found that plant NAOGAT clade include other known monofunctional paralogs. Structural analysis and comparison with other known paralogues indicate electrostatic potential shift upon acetylation, suggesting importance of the C‐terminal region in substrate turnover.

Obtained structural and DSF data suggest that ornithine binding to *Zm*NAOGAT is preferred over glutamate binding. The lingering of the product in an active site may be a sign of product inhibition and perhaps a mechanism for pathway regulation, although further studies are required.

Since plant NAOGATs are the target of mangotoxin action, we performed trials to deduce its possible molecular composition through analysis of protein sequences of the *mbo* and *mgo* operons responsible for mangotoxin production. We have assigned putative roles and ligands for each gene product in each operon. As a result, we were able to provide approximate mass and volume of mangotoxin. Revealing mangotoxin structure and obtaining ability to produce it would allow for further studies regarding its toxicity and potential use as a weed control agent.

## 4 Materials and Methods

### 4.1 Cloning, Protein Production and Purification of NAOGATs

For *At*NAOGAT, *Os*NAOGAT and *Zm*NAOGAT genes starting at codons 36, 39 and 33 respectively were bought from TWIST cloned into pMCSG53 vector. *E. coli* competent cells, BL21 (DE3) Gold (Agilent Technologies) for *At*NAOGAT and Codon+ for *Zm‐* and *Os*NAOGAT were prepared as follows. Desired strain was cultivated overnight on LB medium with appropriate antibiotic at 37°C with shaking. SOB medium was inoculated with 1% of overnight inoculum and cultured until OD_600_ reach 0.3, approximately 3 h. The cells were separated from medium by centrifugation in 4°C and 2000 g. Cells were put on ice and carefully resuspended in 0.4 volume (volume refers to entire SOB medium used in previous step) of wash buffer (10 mM CaCl_2_, 15% glycerol, 30 mM CH_3_COOK, 100 mM MgCl_2_, pH 6) and left for 5 min (still on ice). After incubation cells were once more centrifuged in the same conditions as before and again resuspended on ice in 0.1 volume of storage buffer (75 mM CaCl_2_, 15% glycerol, 10 mM Tris) and left for 5 min on ice. After incubation cells were aliquoted to 1.5 mL tubes and put into −70°C freezer.

Protein production and purification was conducted as in Nielipinski et al. (2024). In short, aforementioned competent cells were transformed, using pMCSG53 vectors via thermal shock method. Inoculum for further protein overexpression consisted of LB medium with 150 µg/mL of ampicillin. Recombinant protein production was conducted using TB‐based auto‐induction medium with 150 μg/mL of ampicillin for 4 h in 37°C and 16 h in 16°C. Bacterial cells were separated by centrifugation, pellets were suspended in binding buffer (50 mM HEPES pH 7.8, 500 mM NaCl, 1 mM TCEP, 20 mM imidazole) and further disintegrated via sonication. Disintegrated cells were centrifuged and separated supernatant was transferred to a column with 3.5 ml of HisTrap HP resin (GE Healthcare) coupled with Vac‐Man (Promega) system. Contaminants were washed during five consecutive wash steps using binding buffer, and the overproduced protein was eluted by the addition of elution buffer (50 mM HEPES pH 7.8, 500 mM NaCl, 1 mM TCEP, 400 mM imidazole). During dialysis, in 20× excess of dialysis buffer (50 mM HEPES pH 7.8, 500 mM NaCl, 1 mM TCEP), His‐Tag was cut using TEV protease and excess imidazole was diluted. Proteins of interest were separated from TEV protease on HisTrap HP resin using binding buffer and further concentrated using Vivaspin 20 concentrators (Sartorius) to 5 mL. Size exclusion chromatography was used simultaneously for buffer exchange (50 mM HEPES pH 7.8, 50 mM NaCl, 100 mM KCl, 1 mM TCEP) and as a final purification step. Protein concentration was determined by spectrophotometric absorbance measurement at 280 nm using MW 44200 Da for *Zm*NAOGAT, 43600 Da for *Os*NAOGAT and extinction coefficient 39420 1/M*cm for both. All values were determined for used constructs using ProtParam (Gasteiger et al. 2005). Concentration of proteins used for crystallisation was ~20 mg/mL.

### 4.2 Crystallisation and Data Collection

Screening for crystallisation conditions was done using BCS screen. In order to obtain crystals of better morphology and quality, stepwise matrix optimisation was carried out, where concentration of every mother liquor component was optimised along buffer type and pH. Crystal of acetylated *Os*NAOGAT with bound ornithine was cocrystallized with 10 mM glutamate and 10 mM CoA utilising hanging drop method with mother liquor containing 0.1 M Bis‐TRIS pH 6.5, 0.15 M MgCl_2_, 30% PEG 3350, additionally crystal was soaked in acetylornithine for 40 min. Crystal of nonacetylated *Zm*NAOGAT bound with ornithine was grown utilising hanging drop method with mother liquor containing 0.1 M HEPES pH 5.5 and 30% PEG 3350, additionally crystal was soaked in mixture of acetylCoA and ornithine for 10 min. Crystal of acetylated *Zm*NAOGAT was cocrystallized with 10 mM acetylglutamate from 25% PEG Smear Broad and 0.1 M citrate‐phosphate buffer pH 5.0. All described drops consisted of 1.5 μL of protein and 1.5 μL of mother liquor. Ethylene glycol was used for cryoprotection of *Os*NAOGAT crystal and PEG 400 was used for *Zm*NAOGAT crystals. After cryoprotection crystals were flash frozen in liquid nitrogen.

Diffraction data of both *Os*NAOGAT and nonacetylated *Zm*NAOGAT were collected in Lund at MAX IV BioMAX. Diffraction data of acetylated *Zm*NAOGAT was collected in Berlin at BESSY beamline BL 14.1. Processing was done using XDS (Kabsch 2010). Data of both *Zm*NAOGAT structures was additionally processed using StarAniso (Tickle et al. 2016) using I/σI cutoff of 3.0 for acetylated and 2.5 for nonacetylated structure. Processing statistics can be found in Table 1.

**Table 1:** Data collection and refinement statistics.

Structure:	*Os*NAOGAT – acetylated complexed with ornithine	*Zm*NAOGAT – acetylated unliganded	*Zm*NAOGAT – nonacetylated complexed with ornithine
**Data collection**			
Beamline	MAX IV, Lund	BESSY BL14.1, Berlin	MAX IV, Lund
Wavelength (Å)	0.729	0.981	0.729
Temperature (K)	100	100	100
Space group	*C* 1 2 1	*P* 2_1_ 2_1_ 2_1_	*P* 2_1_ 2_1_ 2_1_
Unit cell parameters *a, b, c* (Å) α, *β, γ* (°)	168.7, 55.7, 81.3 90.0, 94.0, 90.0	59.6, 80.9, 161.2 90.0, 90.0, 90.0	59.3, 79.8, 160.9 90.0, 90.0, 90.0
Oscillation range (°)	0.1	0.15	0.2
Resolution (Å)	44.76−1.45 (1.54–1.45)	48.04−1.22* (1.29–1.22*)	44.51–1.00† (1.06–1.00†)
Reflections collected/unique	569260/129373	2602824/199020	4102675/304832
Completeness (%)			
Spherical	97.0 (84.3)	86.0 (29.8)	74.8 (21.1)
Ellipsoidal	—	96.1 (70.8)	95.2 (68.7)
Multiplicity	4.4 (3.4)	13.1 (11.9)	13.5 (12.2)
*R*_merge_ (%)	4.0 (33.2)	9.2 (74.7)	8.3 (66.0)
*R*_pim_ (%)	—	2.6 (22.3)	2.3 (26.9)
<*I*/σ(*I)*>	20.3 (3.5)	17.8 (2.3)	17.2 (4.7)
**Refinement**			
*R*_free_ reflections	2100	10020	14705
No. of atoms (non‐H)			
protein	6007	6285	6423
ligands	13	3	77
solvent	881	1237	1152
*R*_work_/*R*_free_ (%)	11.2/13.6	12.1/14.5	12.4/13.8
Mean ADP^a^ (Å^2^)	23.0	14.0	14.0
RMSD from ideal geometry			
bond lengths (Å)	0.01	0.01	0.01
bond angles (°)	1.7	1.9	2.0
Ramachandran statistics (%)			
favoured	98	98	98
allowed	2	2	2
outliers	0	0	0
PDB code	**9TBN**	**9TBO**	**9TBP**

*Note:* Values in parentheses refer to the highest‐resolution shell.

^a^ ADP = atomic displacement parameter.

* Best anisotropic diffraction limit cut‐off was 1.22 and the worst 1.43.

^†^ Best anisotropic diffraction limit cut‐off was 1.00 Å and worst 1.41 Å.

### 4.3 Structure Determination and Refinement

Initial determination for plant NAOGAT structures was done by molecular replacement with *Phaser* (McCoy et al. 2007) from CCP4 suite (Agirre et al. 2023), using *Bacillus halodurans* model (PDB ID: 1VRA (Join Center for Structural Genomics 2005). Both *Zm*NAOGAT and *Os*NAOGAT structures were further rebuilt using *Autobuild* (Terwilliger et al. 2008) from *Phenix* suite (Liebschner et al. 2019). Solved structures were then submitted to a series interchangeable automatic and manual refinement using *Refmac5* (Vagin et al. 2004) and *Coot* (Emsley et al. 2010) respectively. Quality of the refinement was controlled by R_work_ and R_free_ (Table 1). Coordinates and structure factors were deposited in the Protein Data Bank with accession numbers: 9TBN, 9TBO, 9TBP.

### 4.4 Sequence Analysis

Sequence of *Os*NAOGAT was used as an input to search SwissProt database (Famiglietti et al. 2019) using blastP (Camacho et al. 2009). The search yielded 204 sequences with sequence identity between 100% and 26%. Protein sequences were downloaded from UniProt and deduplicated using R (R Core Team 2024) script ElimDupes (https://github.com/pedlefsen/hiv-founder-id/blob/master/ElimDupes.R) – no duplicates were found. Similarly sequences of Ntn hydrolases (InterPro family IPR029055) were downloaded from UniProt (281 sequences), along additional information on active site residue and deduplicated leaving 247 sequences. Motifs in sequences of ArgJ and Ntn proteins were calculated separately using XSTREME (Grant and Bailey 2021) from MEME‐Suite (Bailey et al. 2015). TomTom (Gupta et al. 2007) was used for comparison of motifs calculated in both ArgJ and Ntn proteins. Analysed sequences were then aligned using MAFFT (Katoh 2005) and phylogenetic tree was constructed using IQ‐TREE (Minh et al. 2020)(version 2.2.2.6) with model finder, 1000 repeats of ultrafast bootstrap and 8 cores. Treeviewer (Bianchini and Sánchez‐Baracaldo 2024) and CorelDraw was used for phylogenetic tree visualisation.

In order to assign most probable function of the proteins of *mbo* cluster blastP (Camacho et al. 2009) was used using nr‐clustered and SwissProt databases (Famiglietti et al. 2019). Results for each protein were manually assessed.

### 4.5 Enzymatic Assay

Enzymatic activity of both *Zm*NAOGAT and *Os*NAOGAT was assessed using ninhydrin method as originally described by Chinard (Chinard 1952) using modifications introduced by others (Dénes 1970; Gaitonde 1967; B. H. J. Vogel and Mclellan 1963; H. J. Vogel and Bonner 1956). Specificity of enzyme for reaction in both directions was researched. Assay final volume was 100 μL, each reaction contained 1 μM of enzyme and was conducted for 10 min in 25°C, ninhydrin was prepared as 1.5% solution (w/v) in 6 ml of 0.6 M H_3_PO_4_ and 4 ml of glacial acetic acid. After 10 min 50 μL of ninhydrin solution was added to assay and it was warmed at 95°C for 10 min subsequently. After that, assay plate was cooled by putting on ice for 10 min, after which 50 μL of 2 M NaOH was added to each cell. Assay plate was then left for additional 10 min for colour development in 25°C. Absorbance was measured at 220–780 nm spectrum, data was analysed for 370 nm. Size exclusion chromatography buffer (above) was used for suspending both protein and substrates. To estimate maximum velocity of forward reaction 20 mM of glutamate was used with varying 0‐6 mM concentration of acetylornithine, for reverse reaction 20 mM of ornithine and varying 0–6 mM concentration of acetylglutamate was used. Amount of ornithine produced in a forward reaction and leftover from reverse was calculated by using standard curve, prepared in range of 0‐20 mM of ornithine. Assay for each varying concentration of acetylated substrate was done in quadruple. Reverse reaction product concentration was calculated by subtractions of leftover ornithine from starting 20 mM. Calculation of biochemical parameters was done using R (R Core Team 2024) in Rstudio, with tidyverse (Wickham et al. 2019) for data handling and ggplot2 for plotting. Michaelis‐Menten curve was fitted iteratively using nls() function from base R and outlier rejection was handled by comparing absolute value of a residual of an observation to calculated standard deviation of residuals for all observations multiplied by 1.5, observations above this threshold were deemed as outliers. Minimum two observations per substrate concentration were required to be left as nonoutliers. Next iteration of model fitting and outlier rejection was only allowed if R^2^ of model has improved by at least 0.01 in comparison to previous round, maximum of 5 rounds of iterative model fitting and outliers’ rejection was allowed.

### 4.6 Thermal Shift Assay

Thermal shift assay was performed according to (Kitel et al. 2023). 50 μM *Zm*NAOGAT (*Zea mays*) was preincubated for 2 h at 20°C with 0.2, 1.0 and 5.0 mM ligands, which corresponds to 1:4, 1:20, 1:100 protein:ligand molar ratios. N‐acetylglutamate, N‐acetylornithine, ornithine, glutamate, acetylCoA and CoA were tested. Control and reference samples were apo *Zm*NAOGAT and apo *Zm*NAOGAT treated with DMSO (10% final concentration in the assay). The assay was conducted in 50 mM Tris pH 7.8, 50 mM NaCl, 100 mM KCl, 1 mM TCEP, with minimum four replicates for each sample. Briefly, 20 μL of the preincubated protein:ligand mixture was mixed with 2 μL of SYPRO ORANGE (45×) and 18 μL of the buffer. The assay started at 25°C and the temperature was raised by 0.5°C every 1 min until 95°C. Fluorescence change measured in the FAM channel of BioRad CFX96 Touch Real‐Time PCR was recorded in the range of 25°C–95°C. Data were analysed using R with ligand and concentration combined into composite grouping variable. Normality of data was tested using Shapiro‐Wilk test, homoscedasticity of variance was checked using Levene test. Type II ANOVA with White's adjustment was used to account for lack of homoscedasticity. Melting temperatures of each condition were compared to ligand‐free control using Games‐Howell post‐hoc test using rstatix library. Plots were created using ggplot2 library.

### 4.7 Other Software Used

DSSP (Kabsch and Sander 1983) and Pdbsum1 (Laskowski 2022) was used for secondary structure assignment. Sequence alignments were done using MAFFT (Katoh 2005) with automatic option. Graphical representation of proteins and ligands were done using Chimera (Pettersen et al. 2004). Final images were prepared in CorelDraw. Analysis of surface area and interchain interfaces was done using PISA server. Binding pocket volume analysis was done using kvfinder (Oliveira et al. 2014) in ChimeraX (Meng et al. 2023) with default parameters (probeIn 1.4, probeOut 4.0, removalDistance 2.4), for *Zm*NAOGAT and *Ec*AOD default parameters were changed to better accommodate openness of binding site – probe out was set to 6.5 and removal distance to 1.8. Mass and volume of small molecules was assigned using Python and RDKit using direct method via ComputeMolVolume(). Atom charges were assigned using PDB2PQR (Dolinsky et al. 2007). Electrostatic surface potential was calculated using APBS server (Jurrus et al. 2018). Analysis of motifs was done using Python scripts. Some of the Python scripts were debugged using LLMs.

## Conflicts of Interest

The authors declare no conflicts of interest.

## Supporting information


Supporting File

## Data Availability

The data that support the findings of this study are openly available in Protein Data Bank at https://www.rcsb.org/, reference number 9TBN, 9TBO, 9TBP. The data underlying this article are available in the Protein Data Bank at https://www.rcsb.org/, and can be accessed with the following PDB IDs: acetylated *Os*NAOGAT complexed with ornithine – 9TBN; acetylated *Zm*NAOGAT in unliganded form – 9TBO; nonacetylated *Zm*NAOGAT complexed with ornithine – 9TBP.
