# Supplementary material for: Plant NAOGAT (ArgJ) Structure Reveals Mechanism of Ornithine Biosynthesis, Preference for Acetylornithine and Susceptibility to Mangotoxin
Source: Plant Cell Environ. 2026 Sep 21;49(12):10283–300. doi: 10.1111/pce.70891 (PMC13725253; doi:10.1111/pce.70891)
Supplement: Supplementary file 1 [file PCE-49-10283-s001.docx]

**Plant NAOGAT (ArgJ) structure reveals mechanism of ornithine biosynthesis, preference for acetylornithine and susceptibility to mangotoxin**

**Maciej Nielipinski, Agnieszka J. Pietrzyk-Brzezinska, Aleksandra Twarda-Clapa, Bartosz Sekula**

Institute of Molecular and Industrial Biotechnology, Faculty of Biotechnology and Food Sciences, Lodz University of Technology

SUPPLEMENTARY MATERIALS

*Supplementary Table 1. Calculated melting temperatures determined in thermal shift assay.*

|  | Ligand concentration [mM] | | | |
| --- | --- | --- | --- | --- |
| Ligand / T_m_ [°C] | 0 | 0.2 | 1 | 5 |
| AcGlu | 59.63±0.95 | 61.88±0.25 | 66.25±0.50 | 71.13±0.25 |
| Glu |  | 59.25±0.65 | 59.88±0.48 | 61.50±2.48 |
| AcOrn |  | 60.63±0.25 | 63.75±0.50 | 67.25±0.50 |
| Orn |  | 58.88±0.85 | 59.75±0.87 | 62.50±0.71 |
| AcCoA |  | 60.38±2.25 | 61.38±0.25 | 61.88±0.63 |
| CoA |  | 60.67±1.44 | 59.00±0.91 | 58.88±0.48 |
| DMSO - control |  | 10% DMSO: 57.00±0.50 | | |

*Supplementary Table 2. Proposed function, ligands and assignment of InterPro family of proteins produced as a result of expression of genes of mbo and mgo operons of P. syringae with their closest homologue identified through blastp search against SwissProt and nr clustered .databases. Putative function and product were assigned basing on InterPro family assignment and results of blastp search.*

| Gene | UniProt ID | InterPro families | Putative function of the gene product | Characteristics of the putative product of the catalysed reaction | Closest homologue in SwissProt database | Query coverage against SwissProt hit[%] | Percent identity to SwissProt hit [%] | Closest homologues in clustered nr database (other than hypothetical protein or itself) |
| --- | --- | --- | --- | --- | --- | --- | --- | --- |
| *mboA* | I1VE73 | IPR016084 – haem oxygenase-like, multi-helical | Iron-dependent redox enzyme, possibly aldo/keto reductase | Coenzyme PQQ or structurally similar compound | No homologues found | - | - | Iron-containning redox enzyme family protein from *Streptosporangaceae bacterium* |
| *mboB* | I1VE74 | IPR001041 – 2Fe-2S ferredoxin-type iron-sulfur binding domain  IPR017938 – riboflavin synthase-like beta-barrel | Iron-sulfur cluster binding, oxidoreductase |  | Ribose-phosphate pyrophosphokinase from *Aquifex aeolicus*  Uniprot ID: O67556 | 18 | 41.5 | FAD-binding oxidoreductase from *Pseudomonas* |
| *mboC* | I1VE75 | IPR011761 – ATP-grasp fold  IPR003806 – ATP-grasp fold, PylC-type | Oligopeptide ligase, putative biotin carboxylase | Amide bond | Carnosine synthase from *Gallus gallus* | 67 | 25.1 | Phosphoribosylglycinamide synthetase from *Streptosporangium volaceochromogenes* |
| *mboD* | I1VE76 | IPR003806 – ATP-grasp fold, PylC-type  IPR0011761 – ATP-grasp fold  IPR013815 – ATP-grasp fold, subdomain 1  IPR052032 – ATP-dependent amino acid ligase | Oligopeptide ligase, putative biotin carboxylase |  | Dapdiamide A synthase from *Patoea agglomerans* | 45 | 28.3 | Biotin carboxylase from *Acinetobacter* sp. |
| *mboE* | I1VE77 | - | Amidinotransferase/ guanidine group modification / deiminase | Ornithine, modified arginine or structurally similar compound | No homologues found | - | - | Arginine deiminase family protein from *Pseudomonas* and *Brenneria uluponensis* |
| *mboF* | I1VE78 | IPR050638 – amino acid and vitamin transporters  IPR000620 – EamA domain | Transmembrane transporter, DMT family | compounds containing large cyclic or aromatic groups like sugars, nucleosides or their derivatives | Uncharacterized transporter AF_1552 from *Archaeoglobus fulgidus* | 89 | 27.8 | DMT family transporter from *Pseudomonas* and *Brenneria uluponensis* |
| *mgoA* | Q19AK1 | IPR000873 – AMP-dependent synthetase/ligase domain  IPR009081 – phosphopantetheine binding ACP domain  IPR013120 – fatty acyl-CoA reductase-like, NAD-binding domain | Non-ribosomal peptide synthase, ATP- and NAD-dependent | Peptide, possibly cyclic | Linear gramicidin synthase subunit D from *Brevibacillus parabrevis* | 83 | 30.7 | Non-ribosomal peptide synthetase from *Pseudomonas* |
| *mgoB* | Q19AK3 | IPR016084 - haem oxygenase-like, multi-helical | Iron-dependent redox enzyme, possibly aldo/keto reductase | Coenzyme PQQ or structurally similar compound – polyketide? | No homologues | - | - | DUF3050 domain containing protein from *Pseudomonas* |
| *mgoC* | Q19AK2 | IPR012348 – ribonucleotide-reductase like  IPR025859 – AurF.CmlI | Dimetalic amine oxygenase | nitroaryl | No homologues | - | - | Diiron oxygenase from *Pseudomonas* |
| *mgoD* | Q19AK0 | IPR019587 – polyketide cyclase/dehydrase | Polyketide cyclase/ dehydrase | Polyketide – possibly containing p-nitro amine moiety | No homologues | - | - | SRPBCC family protein from *Pseudomonas*  Polyketide cyclase / dehydrase from *Paucimonas lemoignei* |

*Supplementary Table 3. Calculated approximate binding sites volumes, of ZmNAOGAT, At*OTC, *Tb*ODC, *Zm*GS and *Ec*AOD *known to be inhibited by P.syringae toxins and calculated approximate volumes of inhibitory compounds produced by P. syringae and a known NAOGAT inhibitor p-chloromercuribenzoic acid. Binding sites volumes were calculated using kvfinder in ChimeraX. Molecule volumes were calculated using RDKit in Python3. Volumes are in in Å^3^.*

| Protein | PDB ID | Approximate binding site volume [Å^3^] | Compound | Approximate compound volume [Å^3^] | Compound:binding site volume ratio |
| --- | --- | --- | --- | --- | --- |
| *At*OTC | 8QEU | 400-500 | Phaseolotoxin | 467 | 1:1 |
|  |  |  | Octicidine | 232 | 1:2 |
| *Tb*ODC – with PLP bound | 1QU4 | 450-480 | Phaseolotoxin | 467 | 1:1 |
|  |  |  | Octicidine | 232 | 1:2 |
| *Zm*GS – with ATP bound | 2D3C | 700-800 | Tabtoxin | 261 | 1:3 |
| *Zm*NAOGAT | 9TBP | 500-600 | p-chloromercuri benzoic acid | 158 | 1:3-4 |
| *Ec*AOD | 7RSF | 500-700 | - | - | - |

Supplementary Table 4. Calculated approximate volumes (in Å^3^) of biogenic aminoacids. Volumes were calculated RDKit using Python.

| Amino acid | Approx. volume [Å^3^] | Amino acid | Approx. volume [Å^3^] |
| --- | --- | --- | --- |
| Alanine | 87.34 | Leucine | 137.86 |
| Arginine | 169.99 | Lysine | 150.66 |
| Asparagine | 119.70 | Methionine | 140.20 |
| Aspartic acid | 116.29 | Phenylalanine | 159.30 |
| Cysteine | 105.98 | Proline | 127.08 |
| Glutamine | 136.64 | Serine | 95.86 |
| Glutamic acid | 133.32 | Threonine | 112.94 |
| Glycine | 70.23 | Tryptophan | 187.18 |
| Histidine | 140.22 | Tyrosine | 168.13 |
| Isoleucine | 138.13 | Valine | 121.08 |

Supplementary Table 5. Masses, volumes and mass to volume ratio of amino acids and biological ligands of analyzed proteins. Masses were derived from sequences, volumes were calculated RDKit using Python

| Name | SMILES | Mass [Da] | Volume [Å3] | mass/volume [Da/Å3] |
| --- | --- | --- | --- | --- |
| Alanine | N[C@@H](C)C(=O)O | 89.05 | 87.34 | 0.98 |
| Arginine | N[C@@H](CCCNC(N)=N)C(=O)O | 174.11 | 169.99 | 0.98 |
| Asparagine | N[C@@H](CC(=O)N)C(=O)O | 132.05 | 119.70 | 0.91 |
| Aspartic acid | N[C@@H](CC(=O)O)C(=O)O | 133.04 | 116.29 | 0.87 |
| Cysteine | N[C@@H](CS)C(=O)O | 121.02 | 105.98 | 0.88 |
| Glutamine | N[C@@H](CCC(=O)N)C(=O)O | 146.07 | 136.64 | 0.94 |
| Glutamic acid | N[C@@H](CCC(=O)O)C(=O)O | 147.05 | 133.32 | 0.91 |
| Glycine | NCC(=O)O | 75.03 | 70.23 | 0.94 |
| Histidine | N[C@@H](CC1=CN=CN1)C(=O)O | 155.07 | 140.22 | 0.90 |
| Isoleucine | N[C@@H](C(C)CC)C(=O)O | 131.09 | 138.13 | 1.05 |
| Leucine | N[C@@H](CC(C)C)C(=O)O | 131.09 | 137.86 | 1.05 |
| Lysine | N[C@@H](CCCCN)C(=O)O | 146.11 | 150.66 | 1.03 |
| Methionine | N[C@@H](CCSC)C(=O)O | 149.05 | 140.20 | 0.94 |
| Phenylalanine | N[C@@H](CC1=CC=CC=C1)C(=O)O | 165.08 | 159.30 | 0.97 |
| Proline | N1CCCC[C@H]1C(=O)O | 129.08 | 127.08 | 0.98 |
| Serine | N[C@@H](CO)C(=O)O | 105.04 | 95.86 | 0.91 |
| Threonine | N[C@@H](C(O)C)C(=O)O | 119.06 | 112.94 | 0.95 |
| Tryptophan | N[C@@H](CC1=CNC2=CC=CC=C12)C(=O)O | 204.09 | 187.18 | 0.92 |
| Tyrosine | N[C@@H](CC1=CC=C(O)C=C1)C(=O)O | 181.07 | 168.13 | 0.93 |
| Valine | N[C@@H](C(C)C)C(=O)O | 117.08 | 121.08 | 1.03 |
| tabtoxin | C[C@H]([C@@H](C(=O)O)NC(=O)[C@H](CC[C@@]1(CNC1=O)O)N)O | 289.13 | 261.74 | 1.1 |
| phaseolotox | C[C@H](C(=O)N[C@H](CCCCN=C(N)N)C(=O)O)NC(=O)[C@@H](CCCNP(=O)(N)NS(=O)(=O)O)N | 531.20 | 467.52 | 1.1 |
| octicidine | C(C[C@@H](C(=O)[O-])[NH3+])CNP(=O)(N)NS(=O)(=O)[O-] | 289.04 | 232.26 | 1.2 |
| ornithine | C(C[C@@H](C(=O)O)N)CN | 132.09 | 133.57 | 1.0 |
| carbamoylp | C(=O)(N)OP(=O)(O)O | 140.98 | 102.85 | 1.4 |
| acetylornithine | CC(=O)N[C@@H](CCC[NH3+])C(=O)[O-] | 174.10 | 170.63 | 1.0 |
| acetylglutamate | CC(=O)NC(CCC(=O)O)C(=O)O | 189.06 | 170.41 | 1.1 |
| citrulline | C(C[C@@H](C(=O)O)N)CNC(=O)N | 175.10 | 166.22 | 1.1 |
| putrescine | C(CCN)CN | 88.10 | 104.40 | 0.8 |
| glutamine | C(CC(=O)N)[C@@H](C(=O)O)N | 146.07 | 136.86 | 1.1 |
| PCMB | C1=CC(=CC=C1C(=O)O)[Hg]Cl | 357.97 | 158.30 | 2.3 |
| ATP | C1=NC(=C2C(=N1)N(C=N2)[C@H]3[C@@H]([C@@H]([C@H](O3)COP(=O)(O)OP(=O)(O)OP(=O)(O)O)O)O)N | 507.00 | 369.11 | 1.4 |
| PLP | CC1=NC=C(C(=C1O)C=O)COP(=O)(O)O | 247.02 | 200.76 | 1.2 |

Supplementary Figure 1. Activity of OsNAOGAT with ligand different acetylated ligands – acetylornithine (blue) and acetylglutamate (purple). Activity of enzyme is expressed as percent of calculated theoretical maximal velocity of reaction. Theoretical maximal velocity is calculated by fitting the Michaelis-Menten equation to experimental data using R. Goodness of fit is expressed as R^2^. Calculated R^2^ for reaction with acetylornithine and acetylglutamate were 0.99 and 0.93 respectively.
